# Successful treatment of emphysematous cholecystitis by laparoscopic surgery

**DOI:** 10.1093/jscr/rjab080

**Published:** 2021-03-25

**Authors:** Hitoshi Funahashi, Tetsuya Komori, Naoki Sumita

**Affiliations:** Division of Surgery, JA Mie Inabe General Hospital, Inabe, Japan; Division of Surgery, JA Mie Inabe General Hospital, Inabe, Japan; Division of Surgery, JA Mie Inabe General Hospital, Inabe, Japan

## Abstract

Emphysematous cholecystitis (EC) is a severe and rare variant of acute cholecystitis characterized by ischemia of the gallbladder wall with gas-forming bacterial proliferation. Open cholecystectomy is traditionally the gold standard approach to treatment due to difficulty in isolating Calot’s triangle in the setting of intense inflammation. We present a case of EC successfully and safely treated by laparoscopic surgery.

## INTRODUCTION

Emphysematous cholecystitis (EC) is critical variant of acute cholecystitis in which gas-forming bacteria infect the gallbladder wall [1, 2]. It is associated with high mortality rate due to gangrene and perforation of the gallbladder [1]. Clinical symptoms of EC are similar to those of acute cholecystitis, with fever and right hypochondrial pain being the most common [1]. The mainstay for diagnosis is the presence of gas within the gallbladder lumen or gallbladder wall on imaging [3]. EC requires emergent intervention with open cholecystectomy being the traditional approach [2]. Laparoscopic surgery is difficult due to the anatomical distortion caused by severe inflammation. However, we herein present a case of successful EC treatment via laparoscopic cholecystectomy.

## CASE REPORT

**Figure 1:**
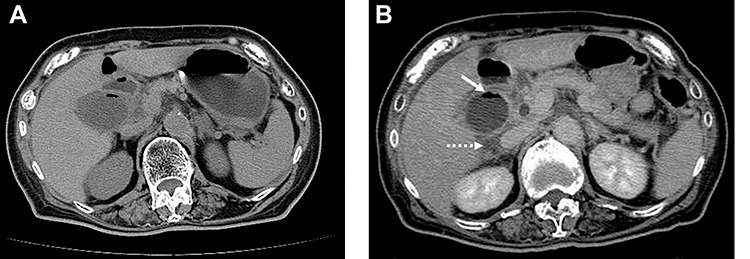
On admission, abdominal computed tomography (CT) scan showed a gas-fluid level in the gallbladder and surrounding fatty infiltration with ascites (**A**). On hospital day 2, abdominal CT revealed increased gas within the gallbladder (**B**, white arrow) and the development of pericholecystic fluid (B, dot arrow).

A 79-year-old female with a history of a cerebral infarction was admitted to our hospital due to nausea, epigastric pain and watery diarrhea. Laboratory findings at presentation were notable for elevated white blood cell (WBC) count of 10 400/μl (normal level, <8600/μl) and C-reactive protein (CRP) 0.5 mg/dl (normal level, <0.1 mg/dl). Abdominal computed tomography (CT) revealed that acute cholecystitis with thickening of the gallbladder wall and the gas image within the gallbladder, leading to a diagnosis of EC (Fig. 1A). Despite the diagnosis of EC, her abnormal clinical and laboratory findings were slight. So the decision was made to admit her to the internal medicine department for conservative management with antimicrobial therapy. However, the following day she developed severe abdominal pain with muscular defense. At that time, WBC was 12 500/μl and CRP had further increased to 12.5 mg/dl. Repeat CT revealed increased gas within the gallbladder and newly developed pericholecystic fluid (Fig. 1B), prompting transfer to the surgery department and emergent laparoscopic intervention.

**Figure 2:**
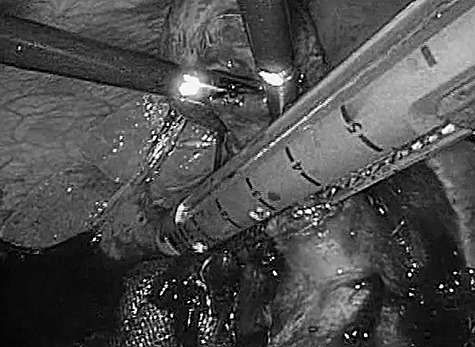
Intraoperative photograph. Significant adhesions and severe infiltration were encountered intraoperatively. The cystic duct and gallbladder artery were treated collectively by an automatic anastomotic device.

Laparoscopic evaluation revealed ascites and severe inflammation of gallbladder without obvious perforation. Despite the presence of serious adhesions, we were able to achieve safe exposure and successful dissection of the cystic duct at the Calot’s triangle (Fig. 2). Afterwards, laparoscopic cholecystectomy, intraperitoneal lavage and drainage were successfully completed without complications or conversion to open surgery.

The patient had an unremarkable postoperative course and was discharged on the ninth postoperative day. Pathological analysis of the resected gallbladder revealed mucosal necrosis and bile cultures grew *Clostridium perfringens*.

## DISCUSSION

EC is a rare and severe variant of acute cholecystitis. The first case of EC was described by Stoltz *et al*. in 1990 [4]. Garcia-Sancho *et al*. [1] reported that the mortality rate approached 25%, with morbidity rates up to 50%. The male-to-female ratio is 7:3 and >40% of EC patients suffer from diabetes mellitus (DM). Most patients who present with EC are over the age of 50 years, and EC is cholecystitis without stone different from common acute cholecystitis [5]. Our patient was elderly, but was female without DM history.

The typical findings of EC are ischemia and gangrene development within the gallbladder. The etiology is thought to be related to vascular compromise of the cystic artery and its branches, the main predisposing factor was vasculitis, atherosclerosis and/or arterial embolic disease. The ischemic changes allowed for mural translocation of gas-forming bacteria and subsequent proliferation, leading to accumulation of gas in the wall and the lumen of the gallbladder. *Clostridium welchii*, *C. perfringens*, *Escherichia coli*, *Klebsiella* and *Streptococci* have been reported in the literature as causative organisms [6]. Our patient did not have apparent vascular disease, but she may have had some risk factors by her prior cerebral infarction. Consistent with the literature, her bile cultures grew *C. perfringens*.

The symptoms of EC are similar to those of acute cholecystitis, including right upper quadrant pain, nausea, vomiting and low-grade fever. Gas around the gallbladder or air within the gallbladder ultimately leads to the correct diagnosis of EC. CT is the most sensitive and specific imaging modality for identification of gas within the gallbladder lumen and biliary ducts [7]. In general, ultrasonography (USG) and plain abdominal radiography can also be used for the diagnosis of EC. However, USG, which depends on the number of air pockets and on localization in the soft tissues, is an operator-dependent and less sensitive technique [7]. CT can clearly show the gaseous halo around the gallbladder and gas-fluid level within it. In addition, CT can detect pericholecystic edema and exclude other differential diagnoses [6]. Therefore, CT is the most useful modality for confirmation of EC diagnosis. In our case, CT revealed thickening of the gallbladder wall and inflammation spreading around the gallbladder. Gill *et al*. [8] divided EC radiographically into three stages according to the distribution of air within the gallbladder and/or the biliary system as follows: Stage I, air in the gallbladder lumen; Stage II, air in the gallbladder wall; and Stage III, air in the pericholecystic tissue. In our patient, gas in the gallbladder lumen was revealed. Therefore, her state was Stage II.

Recently, percutaneous transhepatic gallbladder drainage (PTGBD) has been increasingly performed for EC [9]. However, conservative treatment including PTGBD is not appropriate for EC because of the high risk of gallbladder necrosis and perforation. Definitive treatment for EC is cholecystectomy, traditionally via the open approach [2]. In cases when a laparoscopic approach is attempted, high conversion rates have been reported due to anatomic distortion caused by the significant and severe inflammation that characterizes EC [10]. In addition, it has been recognized that the success of laparoscopic surgery depends on the skill of the surgeon. In 2005, Bouras *et al*. [2] reported that the laparoscopic approach for EC can be considered a safe procedure. Although the inflammation in our patient was severe, particularly within Calot’s triangle, we were able to successfully and safely proceed with laparoscopic surgery for her EC.

Emergency diagnosis and intervention of EC is critical, and a laparoscopic approach can be considered a safe and effective method for EC, although scrupulous care is necessary.

## ACKNOWLEDGEMENTS

None.

## CONFLICT OF INTEREST STATEMENT

The authors declare no conflicts of interest.
